# Estimating changes in lichen mat volume through time and related effects on barren ground caribou (*Rangifer tarandus groenlandicus*) movement

**DOI:** 10.1371/journal.pone.0172669

**Published:** 2017-03-22

**Authors:** Gregory J. M. Rickbeil, Txomin Hermosilla, Nicholas C. Coops, Joanne C. White, Michael A. Wulder

**Affiliations:** 1 Faculty of Forestry, University of British Columbia, Vancouver, British Columbia, Canada; 2 Canadian Forest Service (Pacific Forestry Centre), Natural Resources Canada, Victoria, British Columbia, Canada; University of Alberta, CANADA

## Abstract

Lichens form a critical portion of barren ground caribou (*Rangifer tarandus groenlandicus*) diets, especially during winter months. Here, we assess lichen mat volume across five herd ranges in the Northwest Territories and Nunavut, Canada, using newly developed composite Landsat imagery. The lichen volume estimator (LVE) was adapted for use across 700 000 km^2^ of barren ground caribou habitat annually from 1984–2012. We subsequently assessed how LVE changed temporally throughout the time series for each pixel using Theil-Sen’s slopes, and spatially by assessing whether slope values were centered in local clusters of similar values. Additionally, we assessed how LVE estimates resulted in changes in barren ground caribou movement rates using an extensive telemetry data set from 2006–2011. The Ahiak/Beverly herd had the largest overall increase in LVE (median = 0.033), while the more western herds had the least (median slopes below zero in all cases). LVE slope pixels were arranged in significant clusters across the study area, with the Cape Bathurst, Bathurst, and Bluenose East herds having the most significant clusters of negative slopes (more than 20% of vegetated land in each case). The Ahiak/Beverly and Bluenose West had the most significant positive clusters (16.3% and 18.5% of vegetated land respectively). Barren ground caribou displayed complex reactions to changing lichen conditions depending on season; the majority of detected associations with movement data agreed with current understanding of barren ground caribou foraging behavior (the exception was an increase in movement velocity at high lichen volume estimates in Fall). The temporal assessment of LVE identified areas where shifts in ecological conditions may have resulted in changing lichen mat conditions, while assessing the slope estimates for clustering identified zones beyond the pixel scale where forage conditions may be changing. Lichen volume estimates associated with barren ground caribou movement metrics in an expected manner and, as such, show value for future habitat assessments.

## Introduction

Barren ground caribou (*Rangifer tarandus groenlandicus*) represent one of the most numerous large mammals in arctic and sub-arctic environments [1–3]. Barren ground caribou are commonly grouped into herds based on high fidelity to calving grounds [4–6]. In Canada, barren ground caribou herds are important culturally, ecologically, and economically, as well as for local food security [3, 7, 8]. As foragers, barren ground caribou diets are variable throughout the year depending on forage availability [9]; however, terricholous lichen mats (primarily formed by circumpolar *Cladonia* lichens) form a part of barren ground caribou diet throughout the year [5] and represent the majority of barren ground caribou forage in lean winter months [10]. Terricholous lichens are high in digestible energy, making them excellent (and highly available) winter forage when energy demands are high [9, 10]. Conversely, when animals are adding fat, especially cows during spring and summer, lichen are less desirable owing to their low protein and fat content and availability of alternative forage [10, 11].

Terrestrial arctic and sub-arctic environments are experiencing some of the most rapid climate driven changes on Earth [12–14]. The Arctic tundra is increasing in vegetation greenness [15, 16] (i.e. the Normalized Difference Vegetation Index; used as a proxy for vegetation biomass) and wetness (i.e. the Normalized Difference Moisture Index; used as a proxy for the amount of moisture contained in vegetation biomass) which is likely a result of lengthening growing seasons and warmer summer months [17–19]. Shrub ingress in certain tundra environments is occurring in a rapid fashion, replacing forb/moss/lichen dominated communities with shrub dominated ones which can overtop and out-compete the lower forb/moss/lichen communities [20–22]. Additionally, there is evidence of lichen mat declines in certain tundra regions which have experienced high barren ground caribou densities, where overgrazing and trampling led to reduced lichen abundance [23–25]. Understanding the spatial patterns and temporal dynamics of lichen mats is a critical piece of information for barren ground caribou conservation and management; as such, considerable effort has been focused on this question. Colpaert et al. [26] mapped both terricholous lichen and arboreal lichen resources across Finland while Théau et al. [27] tested multiple methods for classifying lichen land cover across barren ground caribou herd ranges.

Historically, acquiring remotely sensed data in arctic environments has been challenging owing to issues with short growing seasons, snow/ice, and weather. These challenges have been addressed in the past by using coarse spatial resolution imagery (250 m to 1 km spatial resolution) from sensors such as MODIS with rapid (1–2 day) revisit times. Finer spatial resolution satellite programs such as Landsat (30 m spatial resolution) have been less commonly utilised in arctic environments due to the challenge of acquiring images free of cloud (related shadow), haze, and smoke from boreal fires. This challenge is exacerbated by longer revisit times between image acquisitions for Landsat (16 days when only 1 sensor is in operation). However, following the 2008 opening of the United States Geological Survey Landsat archive [28, 29], rapid developments in the application of Landsat TM, ETM and OLI, have taken place [29, 30]. Recent advances in Landsat data processing have included the production of large area annual pixel-based composites [31, 32], and seamless gap free surface reflectance composites of Canada [33, 34]. The developments surrounding Landsat compositing are making assessments of barren ground caribou habitat, including lichen condition, possible at spatial scales appropriate for individual barren ground caribou movement analyses [35].

Recently, Falldorf et al. [36] developed an algorithm (the lichen volume estimator or LVE) for estimating lichen volume from Landsat data, which was successfully applied to Landsat imagery in Norway and is the first ever successful attempt at estimating lichen volume using remotely sensed spectral data. The LVE was built using an in-situ data set of 1345, 50x50 m plots which measured lichen mat volume and were designed to match Landsat spatial resolution (30x30 m) and avoid mixed pixel issues. The LVE is based off two previously developed metrics, the normalized differenced moisture index (NDMI; [37]) and the normalized differenced lichen index (NDLI; [38]), both of which have been previously shown to effectively assess lichen’s spectral signatures. The LVE had an average adjusted R squared of 0.67 using ten-fold cross validation of two dimensional Gaussian regression.

The objectives of this study were to (1) apply the lichen volume estimator developed by Falldorf et al. [36] across the ranges of five barren ground caribou herds in northern Canada from 1985 to 2011, thereby allowing us to (2) assess changes in lichen mat volume both spatially and temporally for five barren ground caribou herds and to (3) assess how changes in lichen mat volume are associated with barren ground caribou movement rates across different seasons. The opening of the Landsat archive allows for the extension of the work of Falldorf et al. [36] both spatially and temporally. Moreover, we have access to a barren ground caribou telemetry data set with over 300 000 individual data points covering five barren ground caribou herds from 2006 to 2011, allowing us to assess how LVE values associate with barren ground caribou movement metrics. We expect an overall decrease in lichen mat volume to have occurred since 1985, especially across the ranges of the more western herds, which have experienced significant expansion of shrubs [20–22]. We also expect that barren ground caribou will exhibit more foraging type behavior in locations with higher lichen mat volume estimates as lichen mats for a critical portion of barren ground caribou diets throughout the year [5, 10].

## Materials and methods

### Study location

**Fig 1:**
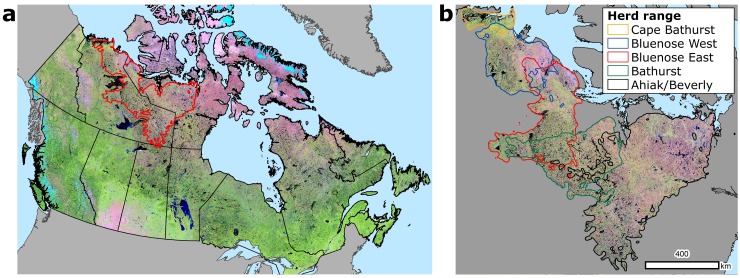
(A) A false-color 2011 Landsat composite proxy image of Canada (Bands 5, 4, 3) with the combined herd ranges of all fiver herds shown in red outline. (B) The five separate ranges of the herds examined here with the same false color image displayed.

Lichen mat volume was estimated across the range of five herds located in the Northwest Territories and Nunavut, Canada. The tundra portion each herd’s range occurs in the northern portion above the boreal treeline. From east to west these herds are known as: The Ahiak/Beverly, Bathurst, Bluenose East, Bluenose West, and Cape Bathurst (see Fig 1 for generalized herd ranges developed using 95% minimum convex polygons). Collectively, the ranges of these herds encompass approximately 700 00 km^2^ of terrestrial habitat.

There is considerable debate regarding the current status of the Beverly herd, estimated at 276 000 individuals (SE = 43 100) in 1994. By 2009, however, the Beverly herd had almost completely abandoned its traditional inland calving grounds south of Garry Lakes [39]. The current gap in knowledge regarding the fate of the Beverly herd is whether the abandonment of its calving grounds was due to a large-scale shift to the traditional calving grounds of the Ahiak (or Queen Maude Gulf) herd beginning in the 1990s [40, 41] or a large numerical reduction in Beverly herd size after which the remnant herd shifted to the Ahiak calving grounds to maintain the advantage of gregarious calving in 2006–2009 [39]. The GPS collar data here do not distinguish between Beverly and Ahiak caribou as the record begins in 2008; the herd range delineated using the collar data is mostly representative of the Beverly range as defined by [40].

### Landsat spectral data

**Fig 2:**
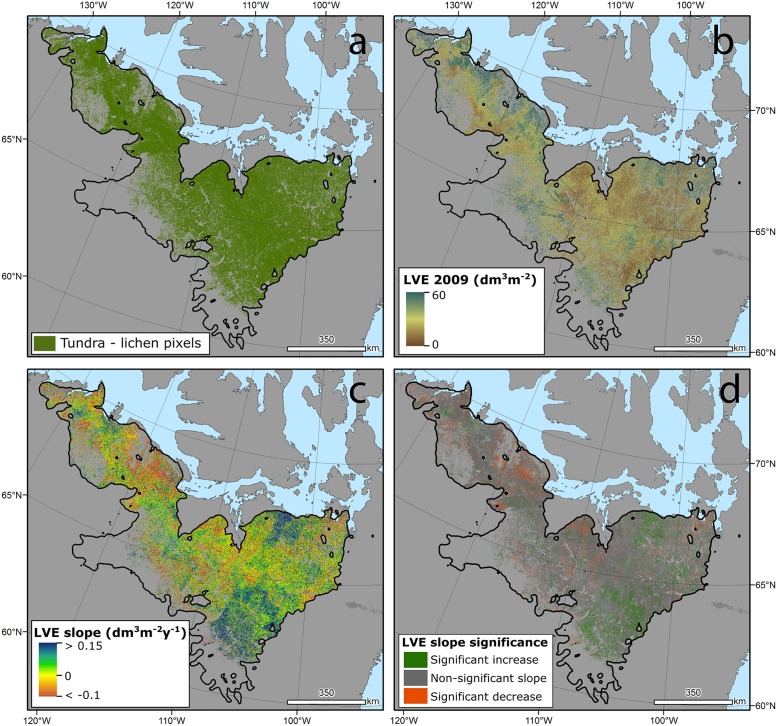
(A) The tundra–lichen map derived from the Canadian Centre for Remote Sensing MODIS land cover data set. (B) 2009 LVE estimates for the five herd ranges. (C) the Theil-Sen slopes estimating change per year for each pixel. (d) significant increases, decreases, and non-significant slopes determined using Mann-Kendall tests.

Following a review of supporting concepts and approaches, White et al. [32] describe a pixel-based image compositing method that identifies best-available-pixels (BAP) using a series of pixel scoring functions appropriate to conditions present in Canada. These functions score each pixel observation based upon (i) Landsat sensor, (ii) acquisition day of year, (iii) distance to clouds and cloud shadows, and (iv) atmospheric opacity (related to presence of haze, smoke). For instance, measures from Landsat-5 are prioritized over Landsat-7; acquisition day of year is prioritized to target August 1 (within a plus / minus 30 day possible acquisition window); with scoring to avoid pixels near clouds or haze. Pixels with the highest scores are selected to produce the BAP image composites for a particular year. Pixels where no observations meet the BAP criteria are labelled as data gaps. These composites were further refined using pixel-level temporal screening to identify noise and remove remaining sources of possible atmospheric contamination (e.g., haze or unscreened clouds). Using the temporal series for each pixel (1984–2012), proxy infill values are generated for data gaps and noisy pixels, following the methods presented in Hermosilla et al. [33], to create gap-free surface reflectance image composites (Fig 2A). This results in Canada-wide, annual, Landsat surface reflectance composites with no spatial or temporal data gaps from 1984 to 2012 [34].

Using this Landsat-derived surface reflectance dataset we computed the lichen volume estimate (LVE) (Fig 2B) on an annual basis by applying the algorithm developed by Falldorf et al. [36]. The LVE product was developed in Norway on an extensive in-situ lichen volume data set on a single Landsat scene (34 225 km^2^). The algorithm employs a multi-dimensional Gaussian curve and two Landsat indices: the Normalized Differenced Lichen Index [38] ([Band 5—Band 2] / [Band 5 + Band 2]; Band 2 representing reflectance in the green portion of the spectrum while Band 5 represents shortwave infrared reflectance) and the Normalized Difference Moisture Index [37] ([Band 4 –Band 5] / [Band 4 + Band 5]; Band 4 representing reflectance in the blue portion of the spectrum). These two indices were combined in the LVE algorithm using the mean parameters calculated by Falldorf et al. [36] through ten-fold cross validation. The scene-wide mean NDMI and NDLI values required in the LVE algorithm were calculated by masking all non-lichen pixels and taking a mean across all combined herd ranges. Lichen tundra pixels were identified using the Canada Center for Remote Sensing’s annual MODIS land cover product, which describes 19 land cover classes across Canada at 250 m spatial resolution from 2001 to 2011 [42]. The 2011 year was used to represent current conditions and tundra classes (tundra grassland and tundra moss/lichen/sparse vegetation classes) were simplified to produce a tundra containing lichen product at 250 m spatial resolution which was then resampled to a 30 spatial resolution to match the Landsat data (Fig 2A). We applied the LVE algorithm across more than 700 000 km^2^ of barren ground caribou habitat (that is, the spatial equivalent of approximately twenty 185 x 185 km Landsat scenes) for every year from 1984 to 2012.

We caution against interpreting the LVE estimates as absolute values of lichen mat volume across our study area, as no in-situ data was available for parameter estimation or validation. However, even using the mean coefficients (which were shown to be stable through cross-validation) established by Falldorf et al. [36] should provide useful information when evaluating the LVE values spatially or through time as potential directional biases resulting in over or under-estimation of actual lichen mat volumes should be consistent. Additionally, Scandinavian and Canadian tundra ecosystems share dominant lichen types (genus *Cladonia* primarily) which are expected to have largely similar spectral reflectance characteristics in the different environments and both indices employed in the LVE (NDMI and NDLI) have been shown to be associated with lichen cover. Despite this, we recommend treating our application of the LVE as a relative index of lichen mat condition rather than a direct, absolute, measurement of volume.

### Barren ground caribou telemetry data

Depending on the herd, GPS collar data collection was initiated between 2006 and 2008 and continued to 2012 matching our most recent composite Landsat data. GPS telemetry data provides latitude/longitude coordinates as well as a time stamp for each data point. GPS error in Canada’s north is typically less than 20 m (less than one 30 x 30 m Landsat pixel). Animals were used here if they had at least one complete year of tracking and a minimum of three GPS points per day, resulting in a maximum time step of eight hours (some individuals were tracked at five hour time steps). In total, 258 animals (223 cows and 35 bulls) have been tracked across all five herds (resulting in 325 388 GPS data points), with a maximum of 66 individuals for the Bluenose West herd and a minimum of 30 individuals for the Ahiak/Beverly herd.

Animal behaviour has been described using proxy measures derived from animal tracking data [43–46]. Foraging behavior in animals such as barren ground caribou can often be recognized by reduced movement speeds as compared to more rapid movement when the animal is travelling [43, 47]. There are numerous other factors that should also be considered which can affect barren ground caribou movement patterns (see Hebblewhite and Haydon [48] for a general summary of GPS collar limitations), including but not limited to: predator and insect avoidance, rutting, and landscape features such as impassable terrain and water. By relating velocity (used here due to the use of two different time steps; [44, 45]) to lichen volume estimates we were able to characterize how differing lichen conditions influence barren ground caribou foraging and movement type behaviors.

### Analysis approach

#### Temporal changes in lichen mat volume

Changes in annual LVE were examined using Theil-Sens (TS) non-parametric regression [22, 49, 50] (Fig 2C). The TS analysis calculated all pairwise slopes for the LVE through time for each pixel throughout the image stack (1984–2012), returning the median slope as the estimate of yearly LVE change. TS slopes are less sensitive to outliers than traditional linear regression and, as a result, are becoming more commonly used in time series analyses at the pixel level [18, 50]. Slope significance was determined using non-parametric Mann-Kendall tests (Fig 2D) [22, 51].

#### Local spatial autocorrelation characteristics of temporal changes in lichen mat volume

The Getis-Ord G_i_* statistic [52, 53] was used to assess the spatial dependence of the LVE Theil-Sen’s slope pixels. This allowed us to determine whether pixels representing change in change in LVE over time were spatially organized into local groupings of positive or negative change, and whether or not the degree of spatial clustering observed in the LVI Theil-Sen's slope raster is less than or greater than chance [54]. The G_i_* statistic evaluates a particular pixel against neighbouring pixels and calculates a weighted variate value for the group of pixels which is expressed as a proportion of the sum of the entire data set’s variate value, derived in this case from the entire LVE TS slope raster. This can be compared against an expected value under the assumption of no local spatial autocorrelation [55, 56]. Here, we applied a Queen’s case kernel, with all neighbouring pixels considered, including diagonals. The G_i_* analysis produces "a standardized value which indicates both the degree of spatial autocorrelation in the values of the digital numbers centered on a given pixel and the magnitude of these values in relation to those of the entire image" ([55], p. 2224). This can be considered a z score hence statistical significance is determined using the area under the z curve. The end result is an assessment of all pixels relative to their neighbours to determine whether each pixel is centered in a cluster of more positive or negative values than would likely occur randomly, or conversely, whether the pixel is centered in a cluster displaying no spatial organization.

#### Temporal changes in lichen mat volume across herd ranges

To examine spatial variation in LVE TS slopes, 1000 random samples were taken from each herd’s range ensuring a spatially stratified random sample. This sample was then analyzed in single factor generalized least squares regression (GLS) with a maximum-likelihood iterator, built using the package “nlme” [57] in R [58]. GLS was used, as it allows for the inclusion of spatial autocorrelation functions to account for issues with spatially structured model residuals [59]. The GLS model was assessed for spatially structured residuals using a semi-variogram which did indeed indicate spatial structuring of residuals. A Gaussian spatial correlation structure was added to the GLS model and the model was subsequently re-examined using Likelihood Ratio test with the best model being retained [59]. If significant differences between groups were detected in the final model, a Tukey’s HSD test [60] was used to assess where differences were significant (an alpha value of 0.05 was used for all statistics).

#### Lichen mat volume estimates and barren ground caribou behavior

Barren ground caribou behavior was inferred using velocity estimates derived from the telemetry data which were, in turn, associated with LVE values based on the year in which the telemetry data was collected. Models were built for each season (Seasons were defined using a generalized version of Nagy’s [61] analysis of differing movement rates of barren-ground caribou throughout the year) with the same randomly sampled data set used for the herd range analysis described in the section above. Generalized additive mixed models (GAMM) with a negative binomial link were built to account for over-dispersion in the data. GAMMs allow for non-linear responses to be appropriately estimated while assigning unique animal identifiers as a random effect accounted for repeated measures per animal [35, 46]. All model residuals were examined for spatial autocorrelation using semi-variograms [59]. Since all models displayed evidence of spatially autocorrelated residuals, Gaussian spatial correlation structures were added to the GAMM models [59].

## Results

The Ahiak/Beverly herd was found to have the largest percentage of land with increasing lichen volume estimates (17.1%, Table 1). The Bathurst and Bluenose West were found to have relatively similar percentages of increasing and decreasing lichen containing tundra (7.7 vs. 8.0% and 6.7 vs. 7.2% respectively). The Bluenose East had slightly more than double the percentage of decreasing than increasing lichen containing tundra (10.9 vs. 5.4%) while the Cape Bathurst had almost five times the percentage of decreasing vs. increasing lichen containing tundra (13.0 vs. 2.9%).

**Table 1:** The percentage of significantly increasing, significantly decreasing, and no significant change lichen containing tundra pixels detected by herd range over all lichen containing tundra.

Change	Ahiak/Beverly	Bathurst	Bluenose East	Bluenose West	Cape Bathurst
Increasing	17.0	7.7	5.4	6.7	2.9
Decreasing	4.0	8.0	10.9	7.2	13.0
No Change	79.0	84.3	83.7	86.1	84.1

**Fig 3:**
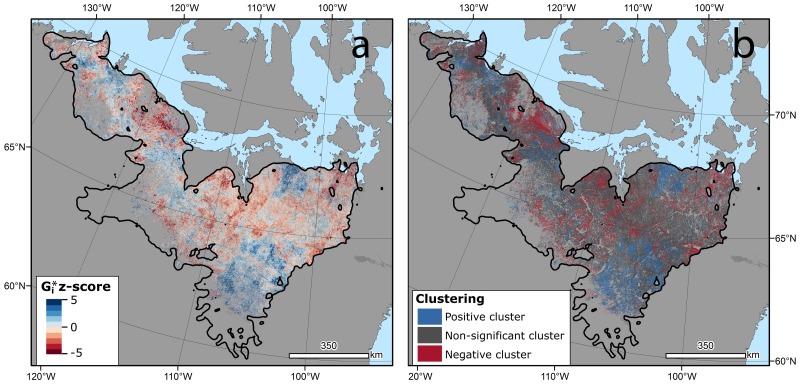
(A) The G_i_* z scores for the LVE Theil-Sen’s slopes. (B) Significant positive, negative, and non-significant G_i_* z score values indicating a pixel was centered in a cluster of positive, negative, or non-significant LVE Theil-Sen’s slope values.

Our analysis of the local spatial autocorrelation contained within the LVE TS slope raster (Fig 3A) revealed that the Bluenose West herd had the largest percentage of its lichen containing tundra pixels centered in clusters of increasing LVE slopes (18.5%; Table 2, Fig 3B), followed closely by the Ahiak/Beverly herd (16.3%). The Cape Bathurst, Bluenose East, and Bathurst herds all had more than 20% (21.0, 20.9, and 20.9% respectively) of their lichen containing tundra pixels centered in clusters of declining LVE slopes.

**Table 2:** The results of the G_i_* analysis, showing the percentage of pixels which were centered in significant clusters of increasing and decreasing LVE TS slope values, as well as pixels displaying no slope clustering, by herd range.

Change Cluster	Ahiak	Bathurst	Bluenose East	Bluenose West	Cape Bathurst
Increasing	16.3	8.4	13.2	18.5	7.4
Decreasing	12.8	20.9	20.9	12.1	21.0
No Change	70.9	70.8	65.9	69.4	71.6

**Fig 4:**
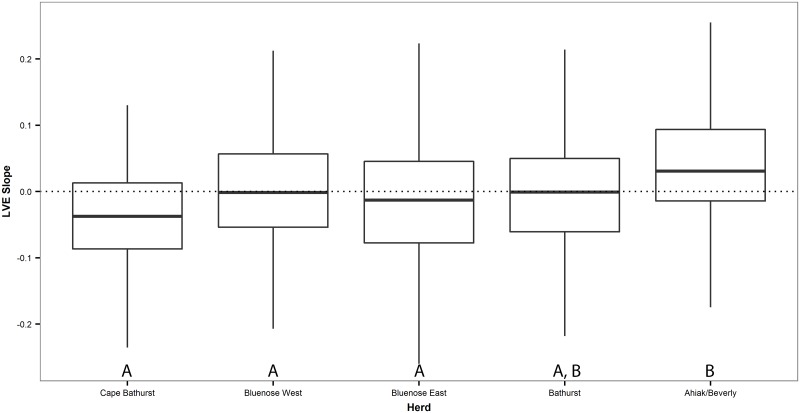
Boxplot of the lichen volume estimate Theil-Sen’s slopes by herd range (west to east). Shared letters indicate non-significant differences.

The LVE slopes by herd range model which incorporated a Gaussian spatial correlation structure out-performed the model lacking one (L. Ratio = 262.84; P < 0.001). A significant difference between classes was detected (F = 4.27; P = 0.002). The Ahiak/Beverly herd range was found to have larger positive slopes than all other herd ranges save the Bathurst (Fig 4).

**Fig 5:**
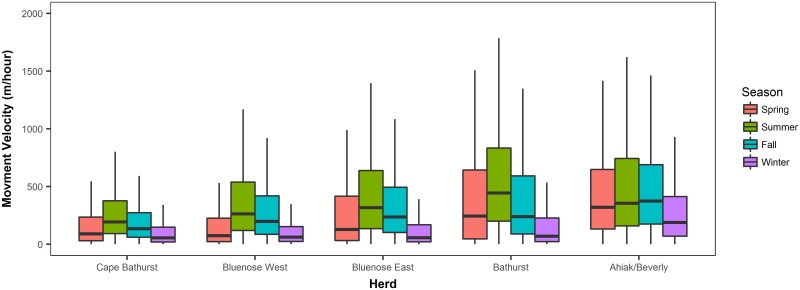
Boxplot of barren ground caribou movement velocities by herd (west to east) and season.

Movement velocities varied by herd and season (Fig 5). Caribou moved fastest in summer (except for the Ahiak/Beverly herds which had slightly higher movement rates in Fall) and slowest in winter across all herds. Fall movement rates were faster than spring movement rates in all herds except the Bathurst. As expected, herds with smaller overall range sizes and lesser distances between their winter habitat and calving grounds (i.e. the Cape Bathurst) tended to move more slowly than herds with large distances between their calving and winter grounds and larger herd ranges (i.e. the Ahiak/Beverly and Bathurst herds).

**Fig 6:**
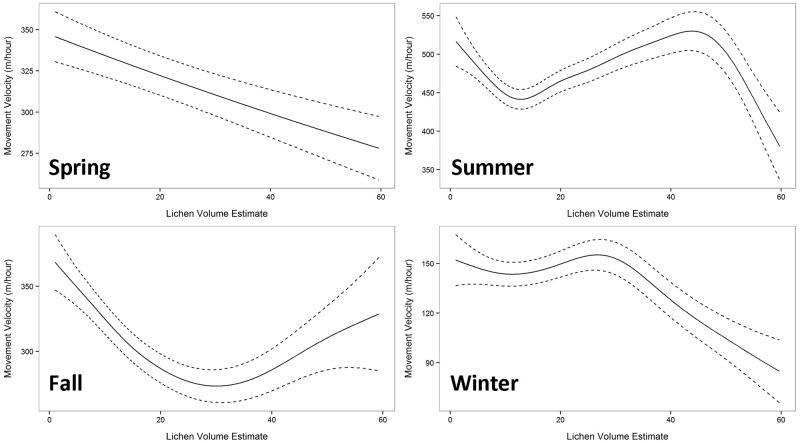
Mean predicted movement velocities (m/hour) predicted using LVE values by season. Dashed lines represent one standard error.

Significant smoothing terms were detected across all seasons for the movement velocity versus LVE GAMMs (Fig 6). A nearly linear negative association between mean movement velocity and LVE was detected in spring (F = 5.121; P = 0.02). A reduction in mean movement velocities was detected at approximately LVE values of 16 and then again after LVE values of 45 in summer (F = 5.92; P < 0.001). Mean movement velocity reached a minimum at LVE values of 25 in fall (F = 10.52; P < 0.001). Mean movement velocities remained relatively constant in winter until LVE values of 25 after which mean movement velocities declined rapidly (F = 3.17; P = 0.02).

## Discussion

In this research, we applied the lichen volume estimator across more than 700 000 km^2^ of barren ground caribou habitat in northern Canada, providing unique information on lichen mat condition and potential barren ground caribou habitat quality. By accessing data available in the Landsat archive and applying novel processing techniques to generate gap-free, time series, reflectance composites we were able to evaluate change in LVE across multiple decades. We detected significant temporal changes in lichen mat volume across the study area and identified significant spatial clusters of positive and negative changes in lichen mat volume. Finally, by incorporating an extensive barren ground caribou telemetry data set we were able to detect subtle changes in barren ground caribou movement rates relative to LVE values.

The Ahiak/Beverly herd experienced significantly larger temporal increases in LVE values when compared with all other herds expect the Bathurst. Conversely, the Cape Bathurst herd experienced the largest overall decline in LVE values, although not statistically different from the Bluenose East, Bluenose West, and Bathurst. The Cape Bathurst herd has declined from 12 516 (SE = 3504) individuals in 1987 to 2427 (a complete herd census therefore no SE estimate is provided) individuals in 2012 (see Boulanger et al. [62] for details of herd count methods). Both Stow et al. [63] and Lantz et al. [20, 21] documented shrub proliferation in the Mackenzie delta/Tuktoyaktuk Coastlands which occur in the western portion of the study area and corresponds with our documented declines in LVE values. This is a product of low productivity lichen/moss/grass communities shifting towards birch- and alder-dominated communities and becoming more productive in the process [22]. Conversely, the continentality of the eastern herd ranges, which results in colder temperatures, may play a role in limiting shrub recruitment [20].

Assessing the LVE TS slope raster for spatial clusters of slope values offers a complementary analysis for assessing LVE changes through time. While the results of the Theil-Sen’s test are pixels occurring in space, the analysis is purely temporal in nature in that the only consideration is one particular pixel. And while this approach is likely of more interest to ecologists examining changing environmental conditions which may be driving changes in lichen mat condition, testing pixels for evidence of local clustering of positive or negative LVE TS slopes allows managers to move beyond the 30x30 m Landsat pixel. As a data capture technology, remote sensing, by nature, partitions the earth’s surface into a grid of regularly sized and shaped cells. When considered independently, ecological and contextual information can be lost. The detailed depiction of landscapes available from the 30 x 30 m spatial resolution of Landsat may be too fine to inform on the larger landscape conditions present. Meaningful spatial aggregation of pixels based upon spatial dependence allows for trend insights that are sized and shaped based upon the conditions present. As an example, spatial clusters of declining LVE values could indicate zones of shrub proliferation [20, 21] or intensive barren ground caribou grazing [24, 25]. In a more applied sense, assessing the landscape by individual Landsat pixel may be a prohibitively small spatial scale for management across herd ranges and testing changes for spatial clustering may identify larger zones of positive or negative change indicating a likely change in barren ground caribou habitat condition.

LVE values were associated with variation in barren ground caribou movement rates in all seasons and these associations changed throughout the year, supporting the need to assess LVE–movement associations by season. Winter movement velocities were lower for all herds, likely a result of individuals moving through snow and having to crater for terricholous lichens. In contrast, summer movement velocities, when cows and calves are moving from calving grounds to summer ranges, were among the highest velocities for each herd.

Summer and winter displayed relatively consistent movement velocities across low and medium LVE values followed by declining movement rates at high LVE values. The summer association did have a reduction in movement velocities that occurred at mean LVE values of 18. When investigated further it was apparent that the summer telemetry points with slow movement rates (velocities less than 450 m/hour) which associated with low LVE values (between LVE = 10 to 25) occurred on calving grounds in the northern portion of each herd’s range. Barren ground caribou use calving grounds primarily as a predator avoidance tactic as these locations are not overly productive or nutritious [64]. This decline in movement at low LVE values is likely associated with calving–barren ground caribou cows with recently dropped calves slow considerably for up to a week or more post calving–and likely does not represent foraging type behavior.

The points during summer and winter where movement velocities slowed consistently allowed us to determine at which point LVE values seem to result improved forage conditions at these times of year (LVE values greater than 45 in summer and 25 in winter). Since lichens make up a greater proportion of barren ground caribou diet in winter [10] it is perhaps not surprising that improved foraging conditions in winter occur at lower LVE values when compared with summer. Spring movement velocities were nearly linearly negatively associated with LVE values, indicating consistent improvement in forage conditions with increasing LVE value. Surprisingly, barren ground caribou movement reached a minimum at intermediate LVE values in fall and movement rates increased at high LVE values. The increased rate of movement through areas with high estimated lichen mat volume is unexpected and unexplained at this point.

Barren ground caribou movement rates are undoubtedly affected by more than lichen availability. Seasonal spring, post-calving, and fall migrations to and from calving grounds can result in elevated movement rates [61], while snow and difficulties accessing terricholous lichens in winter can result in lowered movement rates. Insect harassment, which tends to peak in July, can force individuals to more wind prone areas like eskers and ridges, or north aspect slopes which may have retained snow, and in both cases this can reduce move rates [65, 66]. These factors support our decision to assess movement by season, eliminating a potentially confounding factor in movement variation. We do acknowledge, however, that binning by season will not address factors affecting movement on shorter time frames (i.e. snow depth, insect harassment, calving) nor address spatial variation in these factors.

Access to spatially and temporally expansive Landsat composites allowed for the extension of Falldorf et al.’s [36] assessment of lichen mat volume across five herd ranges for 26 years. Statistically significant temporal changes were detected in lichen mate volume estimates, and these changes varied depending on the herd in question. Temporal LVE TS slopes formed clusters of positive or negative change across the study area, revealing more spatially expansive patterns in increasing and decreasing LVE values when compared with the temporal assessment. Barren ground caribou movement velocities were shown to react to LVE values, with foraging type behavior occurring in a manner consistent with our knowledge regarding barren ground caribou dietary requirement throughout the year; the increase in movement velocities at high fall LVE values being the only exception to this. The lichen volume estimate proved to be a promising method for assessing potential lichen mat volume changes through time across expansive herd ranges and warrants further use. Furthermore, as a relative indicator the LVE showed capacity as a predictor of barren ground caribou behavior; future work describing how landscapes affect how barren ground caribou movement and foraging should incorporate this estimate.

The application of the LVE algorithm here highlights both the strength and weakness of using remote sensing for terrestrial ecosystem assessment in northern environments. The ability to collect synoptic remotely sensed terrestrial data repeatedly through time at a reasonable cost is undoubtedly a benefit to northern research. Conversely, the lack of appropriate in-situ data makes the transition from an index to an estimate challenging. In this case, the spectral responses being measured here led to a valuable assessment of spatial and temporal patterns in lichen mat condition. However, if we wish to fully utilize the LVE and produce lichen mat volume estimates, an in-situ ground campaign with a data collection design appropriate for remotely sensed data needs to be carried out, which is an expensive and challenging endeavour.
